# Monosodium iodoacetate-induced joint pain is associated with increased phosphorylation of mitogen activated protein kinases in the rat spinal cord

**DOI:** 10.1186/1744-8069-7-39

**Published:** 2011-05-20

**Authors:** Younglim Lee, Madhavi Pai, Jill-Desiree Brederson, Denise Wilcox, Gin Hsieh, Michael F Jarvis, Robert S Bitner

**Affiliations:** 1 Neuroscience Research, Global Pharmaceutical Research and Development, Abbott Laboratories, 100 Abbott Park Road, Abbott Park, IL 60064, USA

## Abstract

**Background:**

Intra-articular injection of monosodium iodoacetate (MIA) in the knee joint of rats disrupts chondrocyte metabolism resulting in cartilage degeneration and subsequent nociceptive behavior that has been described as a model of osteoarthritis (OA) pain. Central sensitization through activation of mitogen activated protein kinases (MAPKs) is recognized as a pathogenic mechanism in chronic pain. In the present studies, induction of central sensitization as indicated by spinal dorsal horn MAPK activation, specifically ERK and p38 phosphorylation, was assessed in the MIA-OA model.

**Results:**

Behaviorally, MIA-injected rats displayed reduced hind limb grip force 1, 2, and 3 weeks post-MIA treatment. In the same animals, activation of phospho ERK1/2 was gradually increased, reaching a significant level at post injection week 3. Conversely, phosphorylation of p38 MAPK was enhanced maximally at post injection week 1 and decreased, but remained elevated, thereafter. Double labeling from 3-wk MIA rats demonstrated spinal pERK1/2 expression in neurons, but not glia. In contrast, p-p38 was expressed by microglia and a subpopulation of neurons, but not astrocytes. Additionally, there was increased ipsilateral expression of microglia, but not astrocytes, in 3-wk MIA-OA rats. Consistent with increased MAPK immunoreactivity in the contralateral dorsal horn, mechanical allodynia to the contralateral hind-limb was observed 3-wk following MIA. Finally, intrathecal injection of the MEK1 inhibitor PD98059 blocked both reduced hind-limb grip force and pERK1/2 induction in MIA-OA rats.

**Conclusion:**

Results of these studies support the role of MAPK activation in the progression and maintenance of central sensitization in the MIA-OA experimental pain model.

## Background

Osteoarthritis (OA), recognized as the most common form of degenerative arthritis, is caused by progressive disintegration of articular cartilage, bony overgrowth at the joint margins and synovial proliferation that can result in loss of joint function, disability and chronic pain [1-3]. The use of preclinical pain models to examine the pathogenic mechanisms responsible for OA-induced pain are being utilized for developing more effective therapeutic intervention [4,5]. A commonly used chemical model of OA pain involves intra-articular injection of the metabolic inhibitor monosodium iodoacetate (MIA) in the hind limb knee joint of rats, which disrupts chondrocyte glycolysis through inhibition of glyceraldehyde-3-phosphate dehydrogenase, leading to eventual cell death [6,7]. The progressive loss of chondrocytes following MIA results in histological and morphological changes of the articular cartilage similar to the pathology observed in OA patients [8-10]. In addition, focal bone damage observed with intra-articular MIA injection in rat has been reported to produce peripheral nerve injury as demonstrated by increased expression of the nerve injury marker ATF-3 (activating transcription factor-3) in L5 dorsal root ganglia, consistent with pathogenic changes associated with neuropathic pain [11].

However, analysis of pain behaviors such as weight bearing, tactile allodynia and mechanical hyperalgesia in the MIA-OA model have only recently been established, raising questions as to the appropriate behavioral endpoints for evaluating mechanisms and efficacy of novel analgesics for treating OA [4,7,12,13]. Determining biochemical signaling changes associated with nociceptive behaviors in MIA-injected animals may provide an alternative index of nociception, as well as improved understanding of cellular mechanisms involved in this model of OA pathology.

It has been demonstrated that during the first week following MIA injection, transient synovial inflammation may be the underlying cause of pain, whereas pain sensation in later stages may be due to biomechanical changes affecting the articular cartilage and subchondral bone [7]. Joint inflammation surrounding terminal endings of primary afferent neurons can be sensitized and activated by both normally innocuous and non-painful stimuli (peripheral sensitization) [14,15]. In turn, neurons in the spinal cord also become more responsive to innocuous and noxious stimuli onto the inflamed joint as well as adjacent non-inflamed normal tissue (central sensitization) [15,16]. Together, cellular sensitization in both peripheral and central sensory neurons is believed to be key in the initiation and maintenance of nociceptive transmission in chronic pain [17].

The causes leading to central sensitization of pain can be many-fold. It is known that primary afferent neurons release more transmitters upon stimulation following peripheral sensitization (presynaptic component), and neurons in the spinal cord are more excitable due to changes in receptor sensitivity (post synaptic component) [15]. One possible underling mechanisms for enhanced post synaptic sensitivity is up-regulation of second messenger system activation upon stimulation. Among various second messenger systems associated with pain responses, the family of mitogen-activated protein kinases (MAPKs) is likely candidates for development and maintenance of central pain sensitization. The MAPKs are serine/threonine protein kinases that include extracellular signal-regulated protein kinase (ERK) and p38 [18,19]. In the present study, we investigated the involvement of ERK1/2 and p38 phosphorylation-activation as an index of pain-induced central sensitization in the rat MIA model of osteoarthritis. Evaluating the temporal and activation profile of ERK1/2 and p38 may provide better understanding of disease progression in OA and the role of the MAPKs in development and maintenance of pain-induced central sensitization.

## Results

### MIA-induced pain behavior

**Figure 1:**
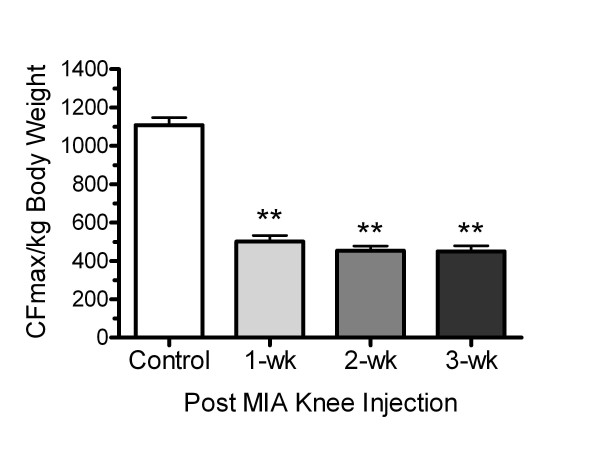
**Hind limb grip force following MIA knee injection in rat**. MIA injection significantly decreased grip force 1, 2, and 3 wk post MIA injection. One-way ANOVA showed a significant treatment effect (F = 101.7, p < 0.0001); post-hoc analysis (Fisher's PLSD) revealed a significant grip force deficit in the 1, 2, and 3 wk-post MIA groups. Grip force strength was expressed as CFmax/kg body weight and represented as mean values (± S.E.M); ** p < 0.01 vs. naïve controls; n = 6/group.

Movement-induced pain behavior was measured using hind limb compressive grip force evaluation in which rats exhibit pain behaviors epitomized by a long-lasting decrement in bilateral compressive hind limb grip force following MIA-induced unilateral knee injury, as previously described [13]. Hind limb grip force was significantly reduced 1, 2, and 3 weeks following MIA injection into the hind-limb knee joint (Figure 1). At each time point, a similar reduction (~ 55%) in grip force was observed in all three OA groups as compared to non-pain controls (ANOVA; f = 101.7, p < 0.0001).

### MIA-induced pERK1/2 immunoreactivity

**Figure 2:**
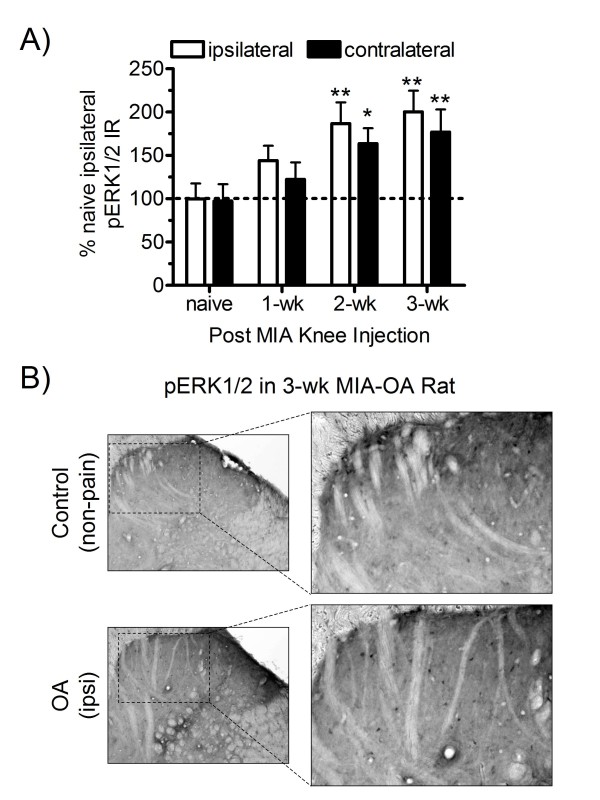
**ERK phosphorylation following MIA injection**. ERK1/2 phosphorylation was increased in both ipsilateral and contralateral dorsal horn spinal cord at 2 and 3 wk following MIA compared to naïve control rats, (A). Phospho-ERK1/2 immunoreactivity (black stained cells) was observed in the upper lamina (hatched box) of the dorsal horn (B) that reached maximal levels at 3-wks as compared to naive (non-pain) controls. An overall ANOVA showed a significant main treatment effect (F = 2.965, p = 0.0134). All data are represented as mean values (± S.E.M) of the percent of ipsilateral naïve control rats; * p < 0.05, ** p < 0.01,  vs. naïve controls (Fisher's PLSD post hoc analysis); n = 6/group.

Spinal cords were harvested (immediately following grip force testing) and immunohistochemically evaluated for changes in MAPK phosphorylation-activation at 1, 2, and 3-wk following intra-articular MIA injection. A significant overall increase in spinal pERK1/2 expression was observed in MIA-OA rats (ANOVA: F = 2.965, p = 0.0134), illustrated in Figure 2. Specifically, increased phospho-ERK1/2 immunoreactivity (black stained cells) was primarily observed in the upper lamina of the ipsilateral dorsal horn (Figure 2B) that reached maximal levels at 3-wks as compared to naive (non-pain) controls (Figure 2A). Similarly, a time-dependent increase in pERK1/2 expression was observed in the contralateral dorsal horn reaching maximal levels in the 3-wk MIA-OA group, albeit to a lesser extent in comparison to the ipsilateral side.

### MIA-induced changes in p38 MAPK immunoreactivity

**Figure 3:**
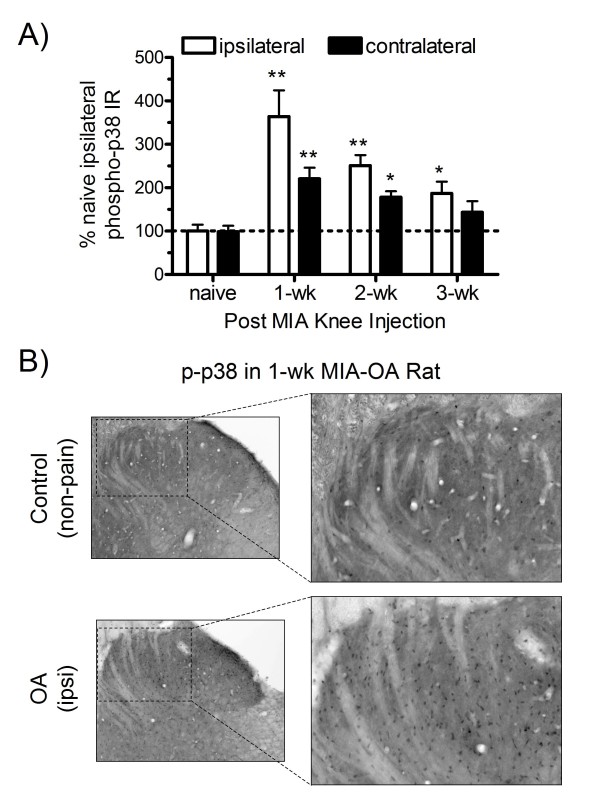
**P38 phosphorylation following MIA injection**. P38 phosphorylation was increased in both ipsilateral and contralateral dorsal horn spinal cord at 1, 2 and 3 wk following MIA as compared to naïve control rats (A). Phospho-p38 immunoreactivity (black stained cells) was observed throughout the upper lamina (hatched box) of the dorsal horn (B) that reached maximal levels at 1-wk and declined thereafter as compared to naive (non-pain) controls (ANOVA: F = 9.686, p < 0.0001). All data are represented as mean values (± S.E.M) of the percent of ipsilateral naïve control rats; * p < 0.05, ** p < 0.01 vs. naïve controls (Fisher's PLSD post hoc analysis); n = 5-7/group.

MIA-treated rats also displayed a significant increase in p38 phosphorylation-activation in the ipsilateral spinal dorsal horn (Figure 3). However, in contrast to pERK, p38 phosphorylation was maximal at post injection week 1 and reduced thereafter (2 and 3 wk), although p-p38 expression remained significantly elevated compared to naïve controls at each time point (Figure 3A). Cellular phospho-p38 immunoreactivity (black stained cells) was observed throughout the dorsal horn lamina (Figure 3B). Similar to ERK, increased p-38 phosphorylation was observed in the contralateral dorsal horn, but to a lesser magnitude in comparison to ipsilateral side that was maximal in the 1-wk group and subsequently declining at 2 and 3 wk following MIA.

### Cellular phenotype of spinal pERK1/2 and p-p38 expressing cells in MIA-OA rats

**Figure 4:**
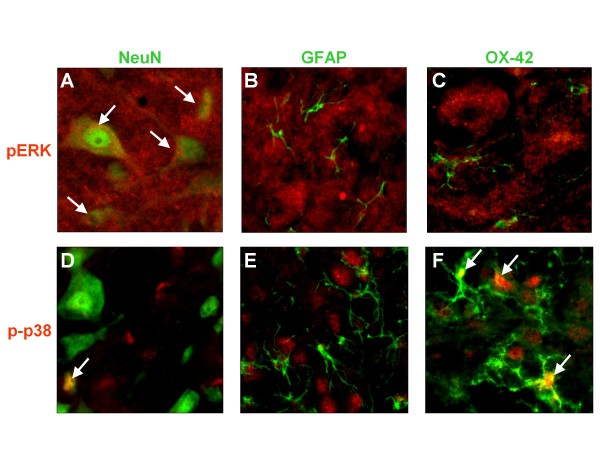
**Expression of spinal pERK1/2 and p-p38 in neuronal and glial cells of MIA-OA rats**. Immunohistochemical double-labeling with the fluorescence dyes Cy2 (red; pERK1.2 or p-p38) and Cy3 (green; NeuN, GFAP, or OX-42) was conducted in lumbar spinal cord sections where orange-yellow fluorescence represents colocalization (white arrow). In the dorsal horn of OA rats 3-wk following MIA, double labeling with anti pERK1/2 (A-C) revealed colocalization with anti-NeuN/neuron (A), but not anti-GFAP/astrocyte (B) or anti-OX-42/microglia (C). Double labeling with anti-p-p38 (D-F) revealed colocalization with anti-NeuN (D), but limited to a subpopulation of small-diameter neurons. Colocalization of anti p-p38 in glial cells was not observed with anti-GFAP (E), but was observed with anti-OX-42 (F).

To determine the cellular phenotype of pERK1/2 and p-p38 expressing cells in the dorsal horn spinal cord of MIA-OA rats, double labeling experiments were conducted with the neuronal, astroglia, and microglia antibodies anti-NeuN, anti-GFAP and anti-OX-42, respectively (Figure 4). Three weeks following MIA injection, ERK1/2 phosphorylation was observed in dorsal horn neurons as evidenced by the colocalization of anti-pERK1/2 and anti-NeuN immunoreactivity (Figure 4A). In contrast, pERK1/2 expression was not observed in either astrocytes (Figure 4B) or microglia (Figure 4C). Conversely, p38 phosphorylation in the spinal dorsal horn was observed in microglia (Figure 4F), but not astrocytes (Figure 4E). In addition, spinal p-p38 expression was also observed in a subpopulation of small-diameter neurons (Figure 4D), in particular at the level of the superficial lamina (1-2).

### MIA-induced changes in OX-42 microglia and GFAP astroglia immunoreactivity

**Figure 5:**
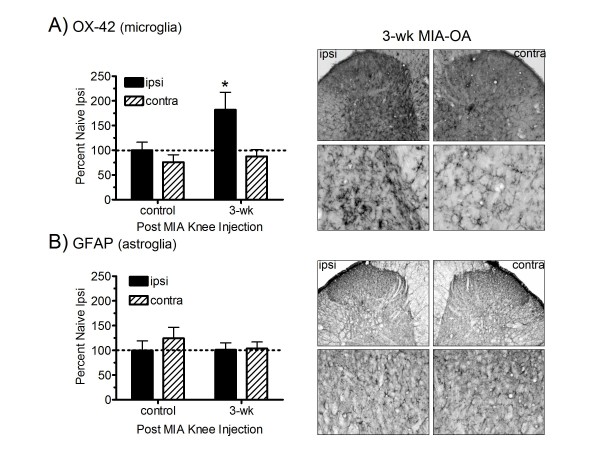
**Spinal glial activation in MIA-OA rats**. Microglia, but not astrocyte, activation was observed in the ipsilateral dorsal horn 3-wk following MIA injection (Figure 5). In 3-wk MIA-treated rats, a significant increase in ipsilateral, but not contralateral, microglia expression was observed in the spinal dorsal horn, as determined by OX-42 (CD11b) immunoassaying, when compared to naive controls (A). In contrast, 3-wk MIA rats exhibited no differences in ipsilateral or contralateral astrocyte expression, as determined by GFAP immunoassaying, when compared to naive controls (B). All data are represented as mean values (± S.E.M) of the percent of ipsilateral naïve control rats; * p < 0.05 vs. naïve controls (Fisher's PLSD post hoc analysis); n = 6/group.

In addition to MAPK expression, spinal microglia activation was examined in OA rats 3-wk following MIA injection (Figure 5). Increased expression of the microglia cell surface marker CD11b was observed in the ipsilateral, but not contralateral, dorsal horn 3-wk following MIA knee injection as compared to naïve (non pain) controls, as measured by OX-42 immunoreactivity (Figure 5A). In contrast, there was no change in ipsilateral (or contralateral) astroglia expression in the dorsal horn 3-wk following MIA injection as compared to controls, as measured by GFAP immunoreactivity (Figure 5B).

### MIA-induced changes in pERK1/2 and mechanical allodynia nociceptive testing

**Figure 6:**
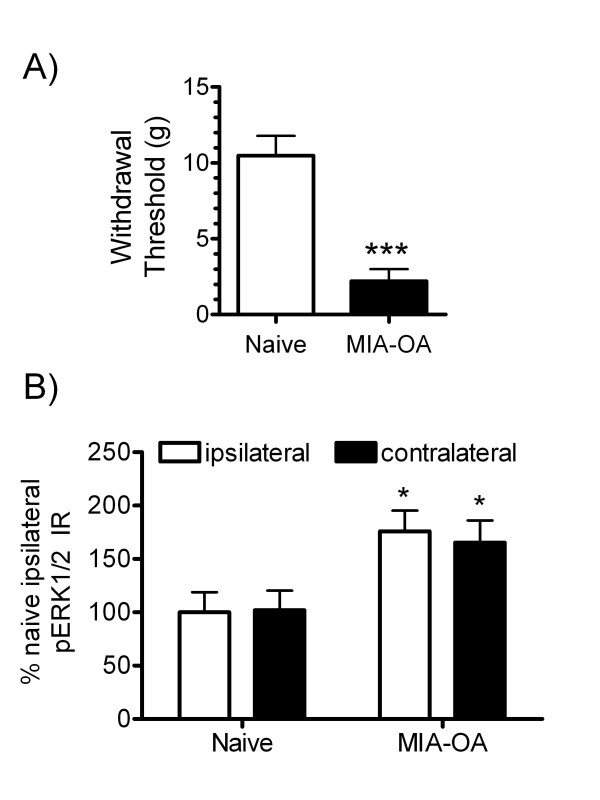
**Comparisons of MIA-induced contralateral paw allodynia to spinal ERK phosphorylation**. Mechanical allodynia, measured as withdrawal threshold (***p < 0.001 vs. naïve control), was observed on the contralateral paw 3-wk following MIA (A). In the 3-wk MIA-treated rats, ERK phosphorylation was observed in both ipsilateral and contralateral dorsal horn spinal when immunohistochemically evaluated immediately following allodynia testing (B). ERK1/2 phosphorylation data are represented as mean values (± S.E.M) of the percent of ipsilateral naïve control rats; ** p < 0.01 vs. naïve controls (Fisher's PLSD post hoc analysis); n = 5-7/group.

To test whether increased MAPK activation observed in the contralateral (to MIA injection) dorsal horn translated to a nociceptive phenotype, mechanical allodynia was assessed on the contralateral paw 3-wk following MIA injection, as measuring grip force does not allow behavioral differentiation between the contra and ipsilateral paw (Figure 6). Withdrawal threshold was significantly reduced (mechanical allodynia) in the contralateral paw 3-wk following MIA as compared to naïve control rats (Figure 6A), a time when reduced grip strength is consistently observed [13]. In the same animals, elevated ERK1/2 phosphorylation was evident in both the ipsilateral and contralateral dorsal horn (Figure 6B). The elevated pERK and mechanical allodynia observed in the contralateral spinal dorsal horn and paw, respectively, of MIA-OA rats supports biochemical translation to a nociceptive phenotype.

### MEK1 inhibitor, PD98059, on MIA-induced pain behavior and pERK1/2 expression

**Figure 7:**
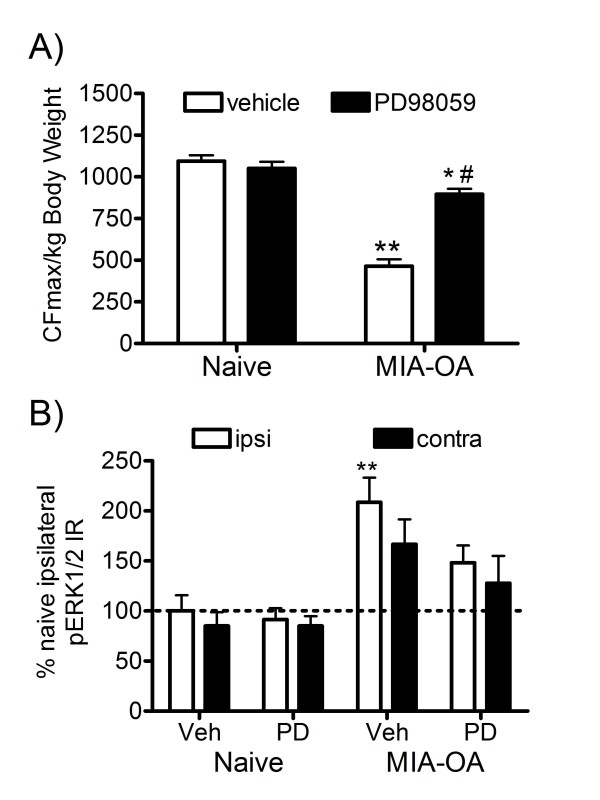
**Effects of MEK (ERK kinase) inhibitor PD98059 on pERK1/2 immunoreactivity in spinal dorsal horns**. Intrathecal administration of PD98059 (10 μg) 30-min before nociceptive behavior assessment attenuated the MIA-induced reduction of grip force strength in 3-wk MIA rats (A). Grip force strength was expressed as CFmax/kg body weight; *p < 0.05, **p < 0.01 vs. naïve controls, # p < 0.05 vs. vehicle alone treated MIA rat; n = 6/group. MIA-OA vehicle-i.t. controls rats displayed a significant increase in spinal pERK1/2 when immunohistochemically processed immediately following grip force testing, whereas PD98059-treated MIA-OA rats did not exhibit the same significant increase (B). ERK phosphorylation data are represented as mean values (± S.E.M) of the percent of ipsilateral naïve control rats; ** p < 0.01 vs. naïve controls (Fisher's PLSD post hoc analysis); n = 6/group.

To examine the functional role of spinal pERK in mediating nociceptive behavior, the MEK (ERK1/2 kinase) inhibitor PD98059 was tested in 3-wk MIA-OA rats. Intrathecal (i.t.) administration of PD98059 30-min before nociceptive behavior assessment significantly attenuated the MIA-induced reduction of grip force strength (Figure 7A). As expected, MIA-OA vehicle-i.t. controls rats displayed a significant increase in spinal pERK1/2 when immunohistochemically processed immediately following grip force testing, whereas PD98059-treated MIA-OA rats did not exhibit the same significant increase (Figure 7B). Together, these results suggest that MIA-induced nociceptive behavior, i.e. reduced grip strength is associated with spinal pERK1/2 phosphorylation-activation.

## Discussion

The use of intra-articular MIA as an animal model of OA has been previously reported to display multiple components of disease progression and symptoms akin to human OA pathology [8-10]. However, demonstration of biochemical changes involving nociceptive signaling in this model are not as well established, in particular markers of central sensitization associated with chronic pain. The present study examined the development and maintenance MAPK phosphorylation-activation in the dorsal horn spinal cord as an index of central pain sensitization in the MIA-OA model. While MIA-injection into the hind limb joint reduced hind limb grip force asymptotically at all three time points tested (post injection week 1, 2, and 3), immunohistochemical evaluation of MAPK activation revealed differential temporal characteristics between pERK1/2 and phospho p38 MAPK. Specifically, pERK1/2 immunoreactivity in dorsal horn of spinal cord, expressed in neurons, but not glia, was gradually increased following MIA injection and reached a significant level at post injection week 2 and 3 compared to naïve control. In contrast, enhanced phosphorylation of p38 MAPK, expressed primarily in microglia, was greatest at post injection week 1 and steadily reduced toward baseline thereafter. In addition, elevated MAPK phosphorylation was observed in the dorsal horn contralateral to the MIA-injected paw, which was accompanied by mechanical allodynia in the contralateral paw of 3-wk MIA-treated rats. Finally, it was demonstrated that i.t. administration of an ERK1/2 inhibitor in 3-wk MIA rats produced antinociception (normalized grip force strength) that correlated with attenuation of spinal pERK1/2 expression. Interestingly, spinal activation of microglia, but not astroglia, was also observed in MIA-treated rats.

It has been suggested that the early, transient synovial inflammation observed in MIA-treated rats may be the predominant cause of initial pain in MIA-OA rats, whereas later pain may result from biomechanical forces affecting the articular cartilage and subchondral bone [7]. It is interesting to speculate that different MAPKs may be involved in stages of OA disease progression, consistent with the temporal-dependent and differential profile of spinal ERK1/2 and p38 phosphorylation in MIA-OA rats observed in the present studies. Although the cellular mechanisms underlying chronic pain syndromes are not well understood, there is accumulating evidence supporting the role of plastic changes involving expression and function of ion channels, receptors and neurotransmitter-peptides in sensory systems responsible for pain transmission (e.g. spinal cord). Among the list of signaling molecules that may regulate the plasticity associated with chronic pain, MAPKs that include ERK and p38 have recently generated much interest [17,20]. As described here, the changes in MAPK phosphorylation-activation observed in MIA-injected rats, a novel finding, support a role of ERK1/2 and p38 in the development and maintenance of pain associated with OA pathology.

Although we are not aware of prior reports examining MAPK expression in MIA-treated rats, or other experimental models of OA, neuropathic pain models involving spinal nerve injury have been well characterized for changes in MAPK phosphorylation involving central and peripheral sensitization. Specifically, activation of spinal ERK1/2 and p38 is induced following peripheral nerve injury in experimental models that includes: L5 spinal nerve ligation (SNL) and chronic constriction injury (CCI) of the sciatic nerve [21-25]. In general, studies conducted in nerve injury models have demonstrated that pERK1/2 activation occurs rapidly and transiently (10 min to 6 hr following nerve injury) in spinal dorsal horn neurons, with subsequent activation in glia cells, both microglia and astrocytes, two to 28 days later. In contrast, p38 induction appears to only occur in microglia, observed one to 14 days following nerve injury.

In the present studies, the diverse temporal profiles of spinal ERK1/2 and p38activation observed in MIA rats may reflect differential MAPK expression by distinct cell types, i.e. neurons and glia, as seen in nerve injury models. Specifically, expression of pERK1/2 was only observed in dorsal horn neurons at 3-wk following MIA, a time point where nociceptive behavior is well established and used in pharmacological antinociceptive testing [13]. In contrast, expression of p-p38 was primarily observed in microglia, but not astrocytes. However, a subpopulation of small diameter neurons in the superficial lamina, also expressed p-p38. It is interesting to note that microglia, but not astroglial, activation was observed in 3-wk MIA-treated rats, consistent with glial p-p38 expression restricted to microglia. Taken together, while there appears to be distinct temporal and biochemical discrepancies between our findings and pain models involving peripheral nerve injury, the pathogenic differences that likely exist between joint and nerve injury may explain these variations.

It is noteworthy that the contralateral spinal dorsal horn also showed a substantial increase in MAPK phosphorylation-activation following MIA injection. Moreover, mechanical allodynia was observed in the contralateral limb, demonstrating the parallel increase in MAPK activity has functional, i.e. pronociceptive consequences. In contrast, spinal MAPK activation reported in nerve injury models is primarily seen in the dorsal horn ipsilateral, but not contralateral, to injury [17]. Interestingly, there have been demonstrations of peripheral-nerve lesions that affect contralateral nonlesioned structures involving signaling via the system of commissural interneurons present in spinal cord and brainstem [26]. The excitatory communications between both sides of the spinal cord have been also demonstrated using electrophysiological techniques; Fitzgrald reported approximately 20% of cells in the substantia gelatinosa of the lumbar spinal cords showed a strong excitatory activation upon tetanic stimulation of the contra lateral sciatic nerve [27]. To our knowledge, the present data is the first demonstration of nociceptive-induced cellular signaling from ipsi to contralateral spinal dorsal horns following MIA injection, a finding that has not been observed in neuropathic peripheral nerve injury models such as SNL and CCI. However, Gao and colleagues recently demonstrated increased bilateral spinal cord expression of the MAPK JNK in the complete Freund's adjuvant model of persistent inflammatory pain [28]. Taken together, results of these studies and those presented here may suggest that the MIA-OA model share biochemical signaling properties of both neuropathic and inflammatory pain states.

It was observed that increased spinal ERK1/2 phosphorylation in 3-wk MIA-OA rats was blocked by the MEK inhibitor PD98059 when examined 30 min following acute intrathecal administration, as would be expected. Moreover, PD98059 treatment partially blocked the pain behavior, reduced grip force strength, observed in MIA-OA rats, supporting the potential involvement in part of ERK1/2 phosphorylation in the dorsal horn spinal cord in mediating nociceptive-induced central sensitization associated with this model of OA. Consistent with our findings, the ability of PD98059 to produce antinociception has been previously demonstrated in various pain models including formalin [29], complete Freund's adjuvant-induced inflammation [30] and CCI [31].

## Conclusions

Evaluation of biochemical pathways associated with central sensitization in animal models of OA has been lacking compared to neuropathic pain models involving peripheral nerve injury. Our current study demonstrates that activation of ERK1/2 and p38 MAPKs in the dorsal horn spinal cord is involved in nociceptive behaviors observed in the MIA-OA model. Furthermore, the ERK and p38 MAPK activation observed, which occurs primarily in neurons and microglia, respectively, displayed different temporal characteristics following MIA injection, suggesting possibly different roles of these MAPKs in development and maintenance of central pain sensitization. Taken together, these findings provide improved understanding of the biochemical relationship of MAPK activation and pain-induced central sensitization in the rat MIA-OA model, and may serve as a mechanistic tool for evaluating novel analgesic agents for the treatment of chronic pain associated with OA.

## Methods

### Animals

Adult male Sprague-Dawley rats (250-300 g, n = 6/group) were used in experiments according to the internal Institutional Animal Care and Use Committee guidelines (Charles River Laboratories, Wilmington, MA). The animals were housed in Association for Assessment and Accreditation of Laboratory Animal Care (AAALAC) approved facilities at Abbott Laboratories in a temperature-regulated environment under a controlled 12-h light-dark cycle, with lights on at 6:00 a.m. Food and water were available *ad libitum *at all times except during testing.

### MIA injection: Osteoarthritic model of pain

Unilateral knee joint osteoarthritis was induced by a single intra articular (i.a.) injection of sodium monoiodoacetate (Sigma, St. Louis, MO) (MIA, 3 mg in 0.05 ml sterile isotonic saline) into the right knee joint cavity under light halothane (Halocarbon Laboratories, River Edge, NJ). Following injection, the animals were allowed to recover from the effects of anesthesia (usually 5-10 min) before returning them to their home cages.

To evaluate antinociceptive behavior and MAPK activation following MIA injection, separate groups of body weight matched Sprague-Dawley rats were injected with MIA on Day 0 (post MIA-injection week 3 group), Day 7 (post MIA-injection week 2 group), or Day 14 (post MIA-injection week 1 group). On Day 21, all MIA-injected animals as well as one group of naïve control animals were subjected to a grip force test. Twenty four hours later, all animals were perfused as described below.

MIA-induced nociceptive behavior at the contra lateral side was examined in a separate experiment. One group of animals was injected with MIA on Day 0 and allowed to recover for 21 days. On Day 21, the MIA-injected animals and one group of naïve control animals received grip force tests. On Day 22, a von Frey test was given to access contra lateral hind paw responses of all animals. Following 24 hour recovery, all animals were perfused, and spinal cords were harvested.

### Intrathecal catheterization and injection of PD98059

The effects of a MEK inhibitor on MIA-induced pain behavior and pERK1/2 expression were evaluated. On the post MIA-injection Day 14, naive control animals, as well as MIA-injected animals received i.t. catheterization as previously described [32]. Briefly, rats were placed under isoflurane anesthesia and mounted onto a stereotaxic instrument using blunt ear bars, which held the animal's head firmly. An incision was made vertically from the dorsal surface of the occipital bone to the base of the skull (2 cm). Tissue was then displaced using a blunt probe so that the alanto-occipital membrane at the base of the skull was clearly seen. An intrathecal PE-10 catheter was inserted through the atlanto-occipital membrane via a small hole in the cisterna magnum. The catheter was then advanced 8.5 cm caudally such that the tip ended in the spinal subarachnoid space around the lumbar enlargement (L4-L6). The catheter was then secured to the musculature at the incision site. The incision was closed with surgical wound clips. The catheter was filled with sterile physiological saline and the ends of the catheter were heat-sealed. Animals with catheters were allowed 1 week of recovery from surgery before behavioral testing. The catheter was subsequently flushed with 10 μl of sterile water to maintain the patency.

On post MIA injection Day 21 (post IT catheterization Day 7), the animals were divided into four groups, one group of MIA-injected animals and one naïve control group animals were injected intrathecally with 10 μg (2 mg/10 ml/1 min/rat) of the MEK (ERK kinase) inhibitor PD98059 dissolved in a vehicle of 10% DMSO/HBC (dimethyl sulfoxide/2-hydroxypropyl-β- cyclodextrin), while remaining groups were injected with the vehicle alone. Thirty minutes after i.t. injection, the animals were subjected to grip force test. Immediately after the behavioral test, the animals were perfused and their spinal cords were harvested.

### Behavioral testing: Hind Limb Grip Force test

Measurements of peak hind limb grip force were conducted by recording the maximum compressive force exerted on the hind limb strain gauge setup, in a commercially available grip force measurement system (Columbus Instruments, Columbus, OH). During testing, each rat was gently restrained by grasping around its rib cage and then allowed to grasp the wire mesh frame (10-12 cm2) attached to the strain gauge. The experimenter then moved the animal in a rostral-to-caudal direction until the grip was broken. Each rat was sequentially tested twice at an approximately 2 min interval to obtain a raw mean grip force (CFmax). These raw mean grip force data were in turn converted to a maximum hind limb compressive force (CFmax) (gram force)/kg body weight for each animal. A group of age-matched naïve animals was added and the data obtained from MIA-injected animals was compared to that of the naïve control group.

### Behavioral testing: mechanical allodynia

Mechanical threshold was measured using calibrated von Frey monofilaments (Stoelting, Wood Dale, IL). Paw withdrawal threshold (PWT) was determined by increasing and decreasing stimulus intensity, and estimated using the Dixon's up-down method [33]. Rats were placed individually in inverted plastic containers (20 × 12.5 × 20 cm) on top of a suspended wire mesh with a 1 cm^2 ^grid to provide access to the ventral side of the hind paws. Rats were acclimated to the chambers for 20 min prior to testing. Monofilaments were presented perpendicularly to the plantar surface of the selected hind paw, and then held in this position for approximately 8 sec with enough force to cause a slight bend in the filament. Positive responses included an abrupt withdrawal of the hind paw from the stimulus, or flinching behavior immediately following removal of the stimulus. A 50% withdrawal threshold was determined using an up-down procedure [34]. The strength of the maximum filament used for von Frey testing was 15 g. A percent maximal possible effect (% MPE) of testing compound was calculated according to the formula: ([compound - treated threshold] - [vehicle - treated threshold])/([maximum threshold] - [vehicle-treated threshold]) × 100%, where the maximum threshold was equal to 15 g.

### Perfusion and tissue harvest

The animals were deeply anesthetized with CO_2 _and perfused through the aorta with buffered saline (3 min, 25 ml/min) followed by 10% formalin (12 min, 30 ml/min). The spinal cords were extracted using hydraulic pressure and post-fixed in 10% formalin and stored in 20% sucrose PBS for overnight before sectioning.

### Immunohistochemistry of free-floating spinal cord sections

After overnight incubation in 20% sucrose-PBS, the lumbar regions of spinal cords containing L3 to L5 were cut on a cryostat (40 μm coronal sections). Prior to sectioning, a knife cut was made through the ventral horn of spinal cords contra lateral to injection site for site specific evaluation of the data. The lumbar sections were immunostained in a free floating system for either anti-phospho p44/42 MAP Kinase (pERK 1/2) antibody (rabbit IgG, 1:700, Cell Signaling #9101), anti-phospho p38 MAP Kinase (Thr180/Tyr182) antibody (rabbit IgG, 1:1000, Cell Signaling # 9211L), anti-CD11b (OX-42) antibody (mouse IgG, 1:500, AbD Serotec), anti-GFAP antibody (mouse IgG, 1:1000, Millipore) using a three-step ABC-peroxidase technique beginning with a 30 min incubation with H_2_O_2_/PBS-Triton solution. Following PBS washes (3X); the sections were incubated with blocking serum (20 min) followed by the primary antibody for an overnight incubation. Twenty hrs later, the sections were washed with PBS (3x), and incubated for 1 hr with a secondary anti-rabbit IgG polyclonal antibody (goat IgG, 1:200; Vector Labs). Immunoreactivity was developed by an ABC (avidin-biotin)-peroxidase reaction using diaminobenzadine (DAB) as a chromogenic substrate. Sections were mounted, cover-slipped, and digitally photographed at 10× (Olympus BX-51 scope with F-View camera) with a shading correction to compensate for uneven illumination. Staining was quantified using an image analysis system (Olympus MicroSuite Five) by measuring number of cells with homogeneous staining of a given antibody in laminae I-III of the spinal dorsal horns.

Double-labeling to determine the cellular phenotype of pERK1/2 and p-p38 expressing cells was carried out by antibody co-incubations of either anti-pERK1/2 or anti-p-p38 with the neuronal marker anti-NeuN, microglia marker anti-OX-42, or astroglia marker anti-GFAP. Specifically, 20 μm free floating lumbar spinal cord sections were blocked for 1-hr in 10% normal donkey serum, at room temperature. Sections were next coincubated with primary antiseras overnight at 4°C that consisted of anti-pERK (rabbit IgG, 1:500, Cell Signaling Technology) with either anti-NeuN (mouse IgG, 1:500, Millipore), anti-GFAP (mouse IgG, 1:1000, Millipore), or anti-OX-42 (mouse IgG, 1:500, AbD Serotec). Similar coincubations using the three cellular antibody markers were also made with anti-p-p38 (rabbit IgG, 1:500, Cell Signaling Technology). Following primary coincubations, sections were washed (3X, 2-hrs) in PBS and co-incubated with secondary fluorescence dye antibodies consisting of donkey-anti-rabbit Cy3 (1:500, Jackson Labs) and donkey anti-mouse Cy2 (1:500, Jackson Lab) for 1-hr at room temperature. Next, sections were washed (2X, 15 min) at room temperature, followed by an overnight wash in PBS at 4°C. Sections were then mounted on slides, dehydrated through series of ETOH (70, 95, 95, 100, 100%) for 2-3 minutes, then cleared in xylene and coverslipped with DPX. Cy2 (green, ~492 nm excitation, ~510 nm emission) and Cy3 (red, ~550 nm excitation, ~570 nm emission) fluorescence microscopy was conducted with an Olympus BX51 fluorescence microscope.

### Statistics

Data were converted to percent change of ipsilateral naïve control and analyzed using one-way or two-way analysis of variance (ANOVA) followed by Fisher's protected least squares difference (PLSD) post-hoc analysis. A p-value < 0.05 was considered statistically significant.

## Competing interests

The authors declare that they have no competing interests.

## Authors' contributions

YL conceived the study, participated in experimental design and interpretation, carried out immunohistochemical experiments and data analysis, and wrote the paper. MP carried out animal surgeries and behavioral pain testing, conducted data analysis for behavioral data, and wrote the paper. JDB carried out immunohistochemical experiments and data analysis. DW carried out immunohistochemical experiments and data analysis. GH participated in experimental design. MJ provided scientific advice for experimental design and manuscript preparation. RSB conceived the project, participated in experimental design and interpretation, and wrote the manuscript. All authors read and approved the text.
